# Supplementary material for: Defining the Immune Checkpoint Landscape in Human Colorectal Cancer Highlights the Relevance of the TIGIT/CD155 Axis for Optimizing Immunotherapy
Source: Cancers (Basel). 2022 Aug 31;14(17):4261. doi: 10.3390/cancers14174261 (PMC9454990; doi:10.3390/cancers14174261)
Supplement: Supplementary file 1 [file cancers-14-04261-s001.zip › cancers-1876917-supplementary.pdf]

Supplementary figure legends

Supplementary Figure S1. Gating strategy for *ex vivo* flow cytometry analysis. (**A**) To analyze the expression of ICs by T-cell populations, the good events (identified by the FlowAI algorithm) were selected using the generated flowAI parameter as well as lymphoid cells with the FSC-A/SSC-A plot. Then, doublets were excluded as well as non-viable cells using the FSC-A/FSC-H and FVS780/FSC-H plots, respectively. To perform supervised analysis, $CD3^+$ cells were selected and for each IC studied, a histogram was generated. To perform unsupervised analysis, a CD4-BUV496/CD8-APC plot was generated to select $CD4^+$ and $CD8^+$ subpopulations. The example gating strategy shown corresponds to the study of a cohort 2 patient. Identical gating strategies were performed for cohorts 1 and 3 with the respective antibodies. (**B**) To analyze the expression of IC ligands by cells present in the tumor microenvironment, the good events (identified by the FlowAI algorithm) were selected using the generated flowAI parameter as well as all living cells with the FSC-A/SSC-A plot. Then, doublets were excluded as well as non-viable cells using the FSC-A/FSC-H and FVS780/FSC-H plots, respectively. An EpCAM-FITC/CD11b-BV421 plot was generated to select $EpCAM^+$ and $CD11b^+$ populations, representing epithelial tumor cells and myeloid cells, respectively, and for each IC ligand studied, a histogram was generated.

Supplementary Figure S2. Study of IC expression by $CD3^+$, $CD4^+$ and $CD8^+$ TILs according to UICC tumor staging system. For each figure, data were analyzed according to tumor stage, with stage I tumors in light pink, stage II tumors in pink and stage III tumors in dark pink. Kruskal-Wallis tests were performed, followed by Dunn’s multiple comparisons tests. (**A**) Frequency of $PD\text{-}1^+$, $TIGIT^+$, Tim-

3^+, $Lag3^+$ and $NKG2A^+$ cells among $CD3^+$ TILs (n=40, cohorts 1, 2 and 3). (**B**) Expression level (median fluorescence intensity, scale in log2) of PD-1, TIGIT, Tim-3, Lag3 and NKG2A on positive $CD3^+$ TILs (n=32, cohorts 2 and 3). (**C**) Proportion of $CD4^+$ and $CD8^+$ cells among $CD3^+$ TILs (n=40, cohorts 1, 2 and 3). (**D**) Cell frequency in each generated cluster of $CD4^+$ and (**E**) $CD8^+$ TILs (n=32, cohorts 2 and 3).

Supplementary Figure S3. Study of IC expression by $CD3^+$, $CD4^+$ and $CD8^+$ TILs according to CMS classification. For each figure, data were analyzed according to CMS classification, with CMS1 tumors in yellow, CMS2 tumors in blue, CMS3 tumors in pink and CMS4 tumors in green. Kruskal-Wallis tests were performed, followed by Dunn's multiple comparisons tests. (**A**) Frequency of PD-1^+, $TIGIT^+$, Tim-3^+, $Lag3^+$ and $NKG2A^+$ cells among $CD3^+$ TILs (n=27, cohorts 1, 2 and 3). (**B**) Expression level (median fluorescence intensity, scale in log2) of PD-1, TIGIT, Tim-3, Lag3 and NKG2A on positive $CD3^+$ TILs (n=22, cohorts 2 and 3). (**C**) Proportion of $CD4^+$ and $CD8^+$ cells among $CD3^+$ TILs (n=27, cohorts 1, 2 and 3). (**D**) Cell frequency in each generated clusters of $CD4^+$ and (**E**) $CD8^+$ TILs (n=22, cohorts 2 and 3).

Supplementary Figure S4. Clustering of $CD4^+$ and $CD8^+$ TILs from cohort 3 depending on their IC expressions. (**A**) FlowSOM tree of $CD4^+$ TILs from cohort 3 tumors (n=12). The background coloring represents meta-clustering and the legends of the star plot and meta-clustering are shown on the left side. (**B**) Heatmap of the median fluorescence intensity of PD-1, TIGIT and Tim-3 markers expressed or not by the 11 clusters identified in the $CD4^+$ TIL population generated from cohort 3 tumors (n=12). (**C**) Cell frequency in each $CD4^+$ TIL cluster generated from cohort 3 tumors (n=12). (**D**) FlowSOM tree of $CD8^+$ TILs from cohort 3 tumors (n=12). The background

coloring represents meta-clustering and the legends of the star plot and meta-clustering are shown on the left side. (**E**) Heatmap of the median fluorescence intensity of the of PD-1, TIGIT, Tim-3, Lag3 and NKG2A markers expressed or not by the 14 $CD8^+$ TIL clusters identified from cohort 3 tumors (n=12). (**F**) Cell frequency in each $CD8^+$ TIL cluster generated from cohort 3 tumors (n=12).

Supplementary Figure S5. UMAP visualization of $CD4^+$ clusters defined by differential IC expression. UMAP visualization of $CD4^+$ clusters of TILs from cohort 2 tumors (n=20) (**A**) and from cohort 3 tumors (n=12) (**B**), with MSS tumors boxed in pale green and MSI tumors boxed in dark blue. UMAP visualization of $CD4^+$ TIL clusters of each tumor in cohort 2 (**C**) and cohort 3 (**D**) patients. Patients targeted by a black arrow are visually considered as outliers.

Supplementary Figure S6. UMAP visualization of $CD8^+$ clusters defined by differential IC expression. UMAP visualization of $CD8^+$ clusters of TILs from cohort 2 tumors (n=20) (**A**) and from cohort 3 tumors (n=12) (**B**), with MSS tumors boxed in pale green and MSI tumors boxed in dark blue. UMAP visualization of $CD8^+$ TIL clusters of each tumor in cohort 2 (**C**) and cohort 3 (**D**) patients. Patients targeted by a black arrow are visually considered as outliers.
