# Supplementary figures and images for: Defining the Immune Checkpoint Landscape in Human Colorectal Cancer Highlights the Relevance of the TIGIT/CD155 Axis for Optimizing Immunotherapy
Source: Cancers (Basel). 2022 Aug 31;14(17):4261. doi: 10.3390/cancers14174261 (PMC9454990; doi:10.3390/cancers14174261)

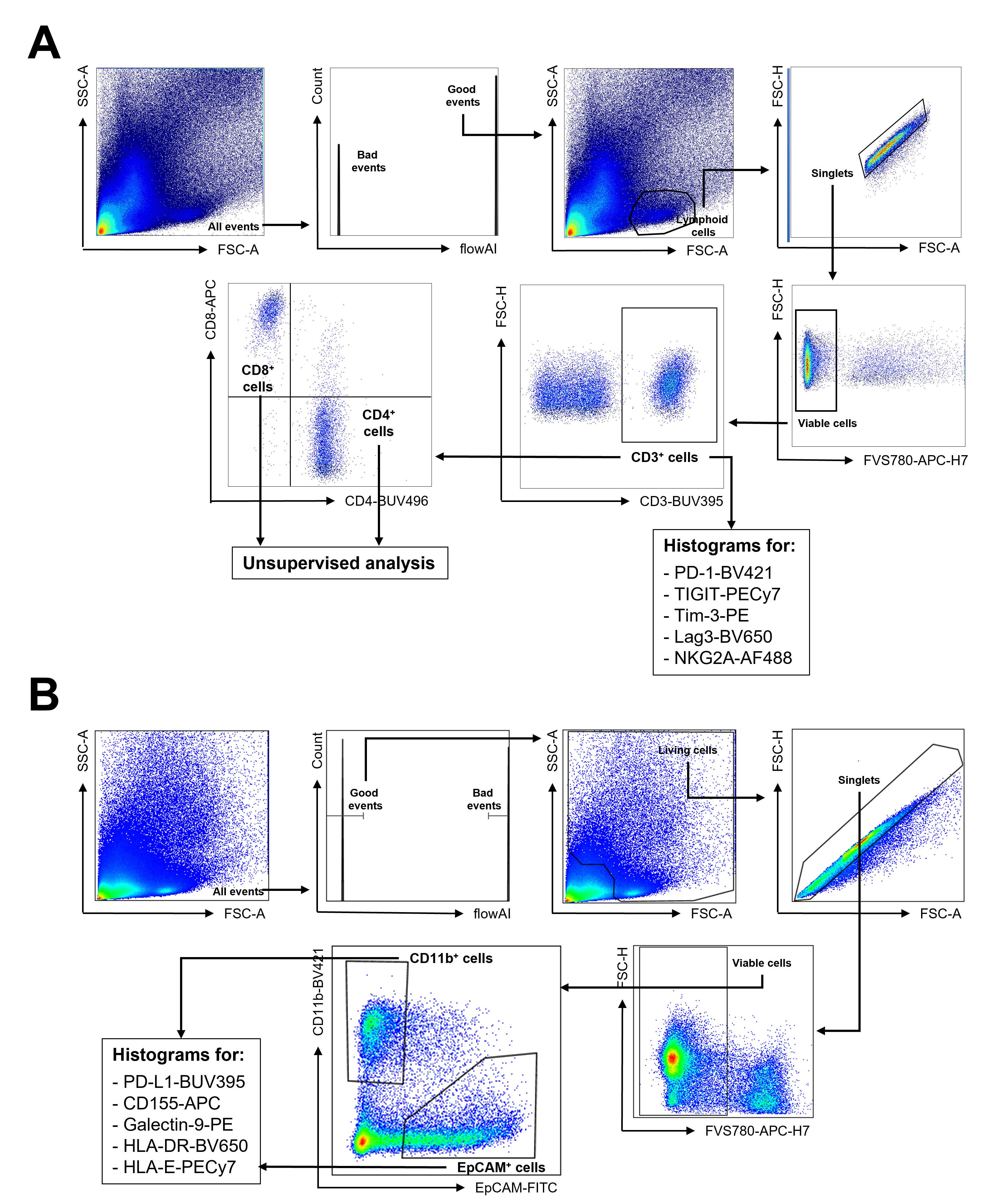

Supplement: Supplementary file 1 [file cancers-14-04261-s001.zip › Figure S1.tif]

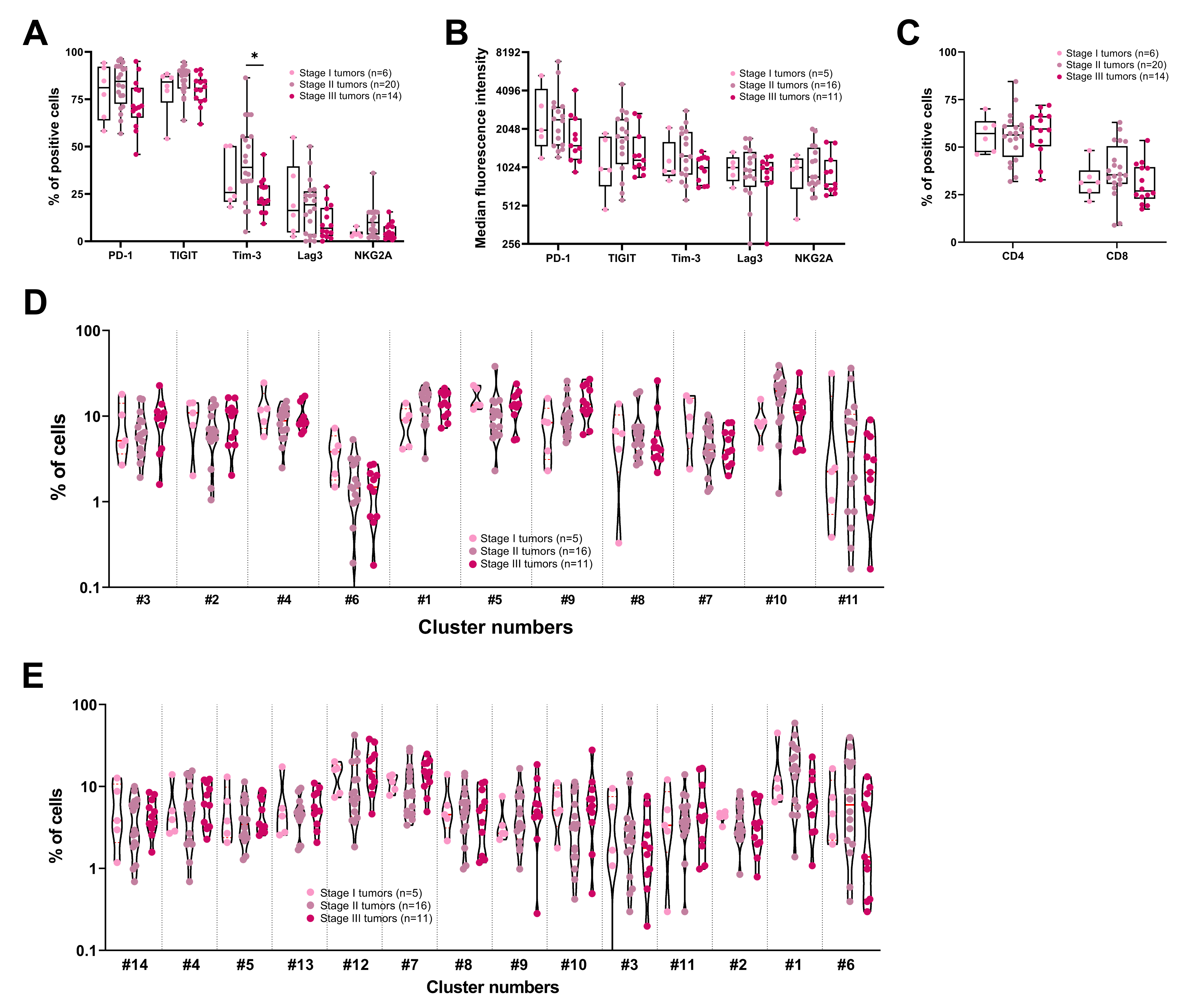

Supplement: Supplementary file 1 [file cancers-14-04261-s001.zip › Figure S2.tif]

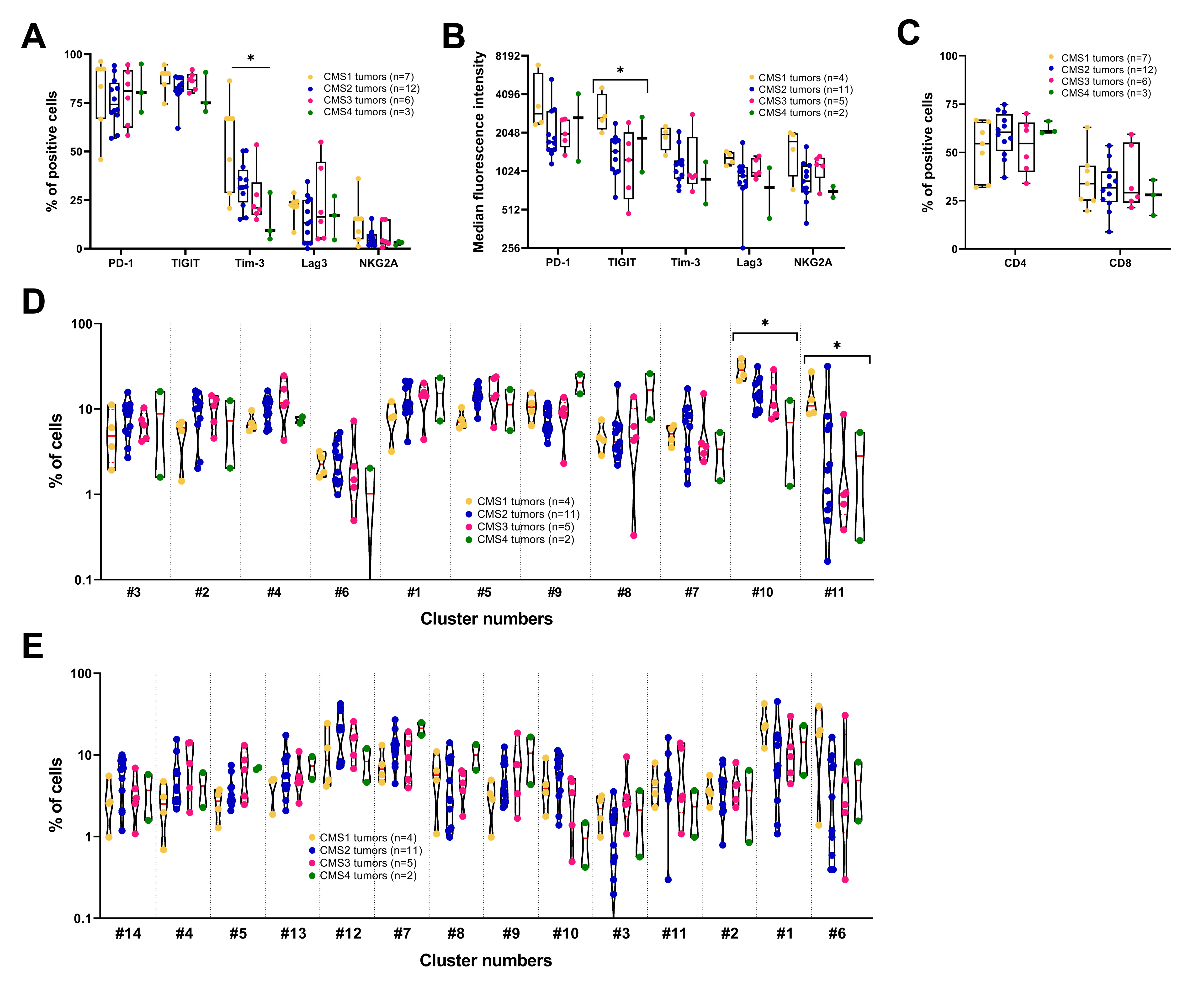

Supplement: Supplementary file 1 [file cancers-14-04261-s001.zip › Figure S3.tif]

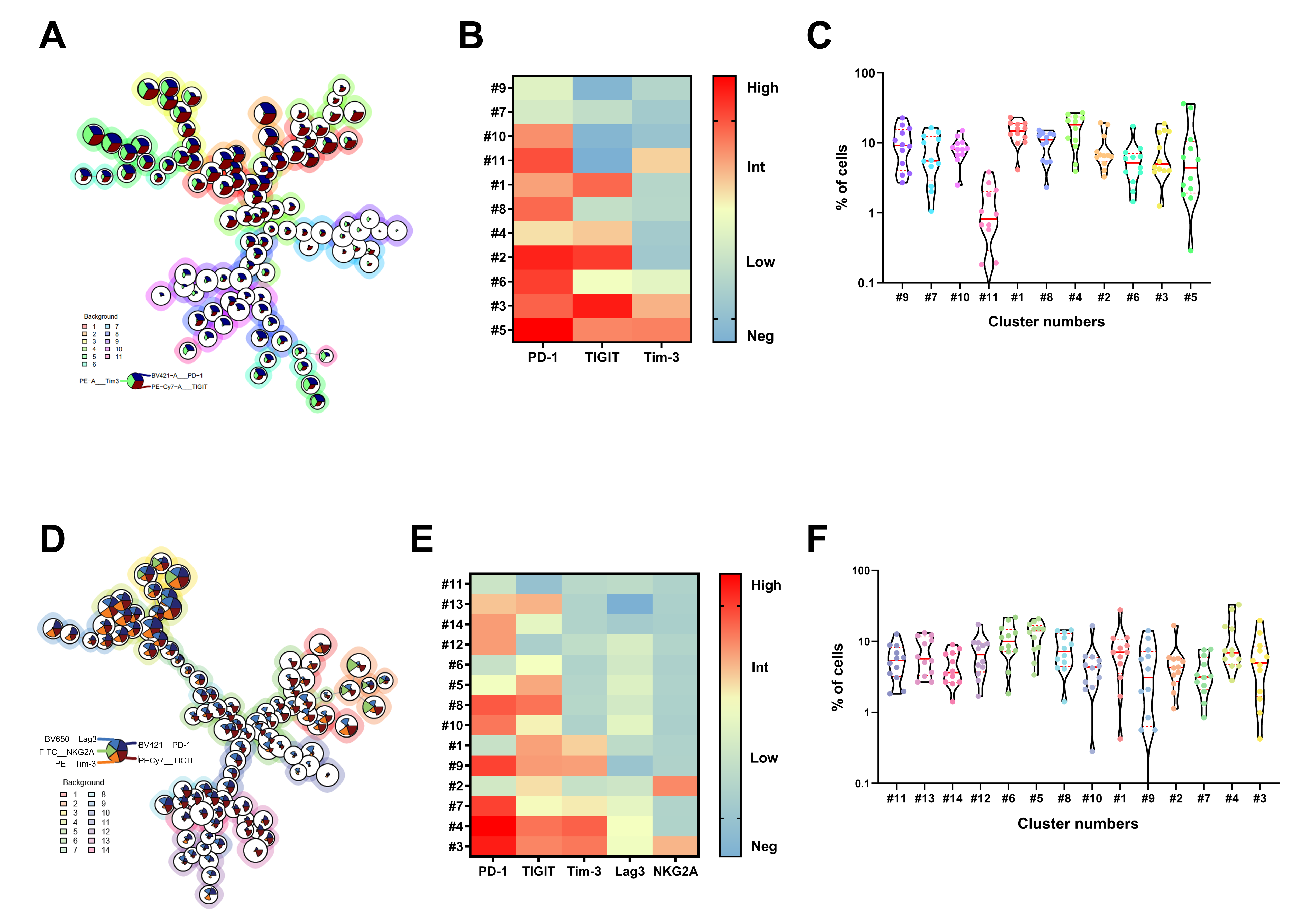

Supplement: Supplementary file 1 [file cancers-14-04261-s001.zip › Figure S4.tif]

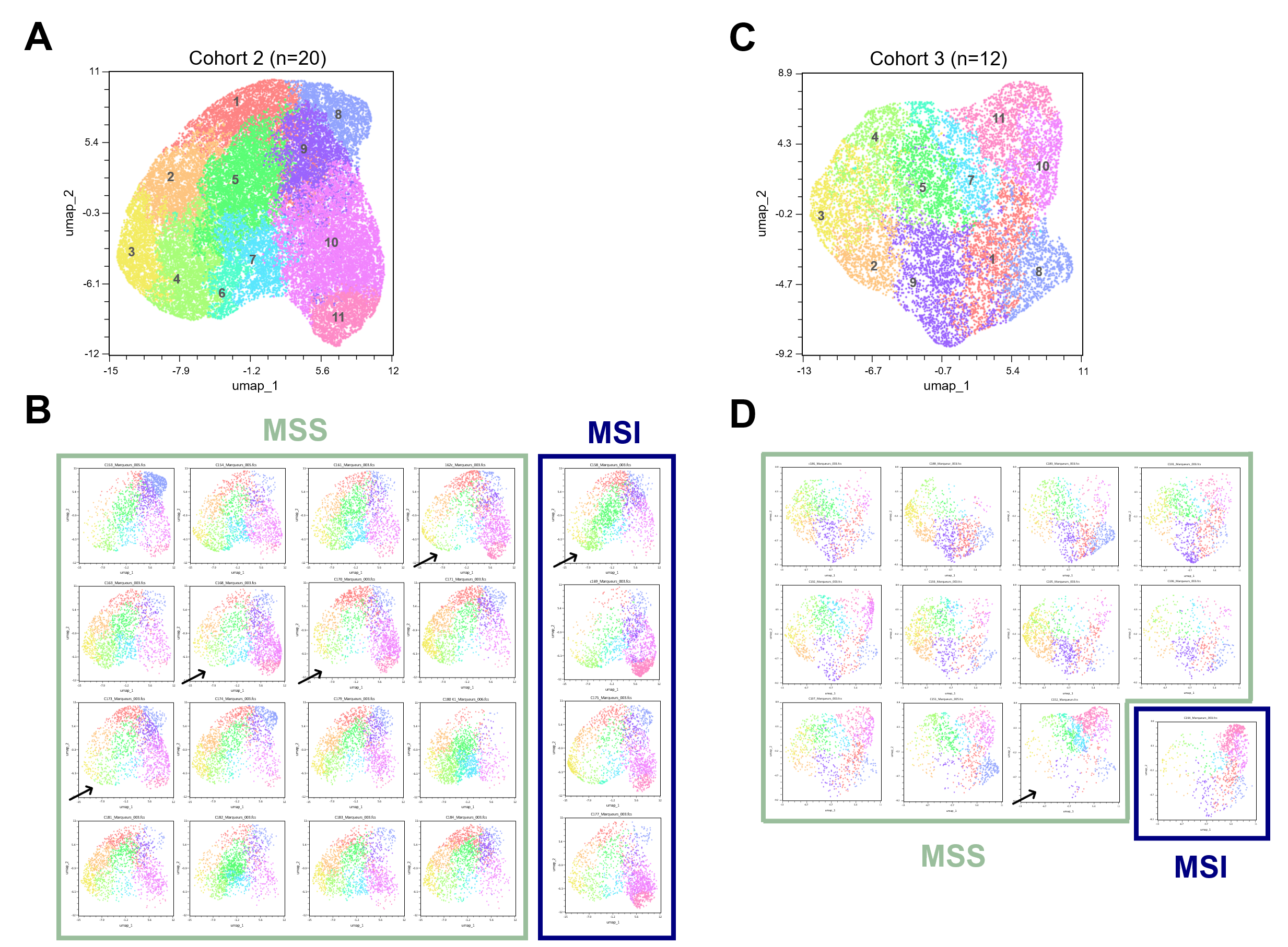

Supplement: Supplementary file 1 [file cancers-14-04261-s001.zip › Figure S5.tif]

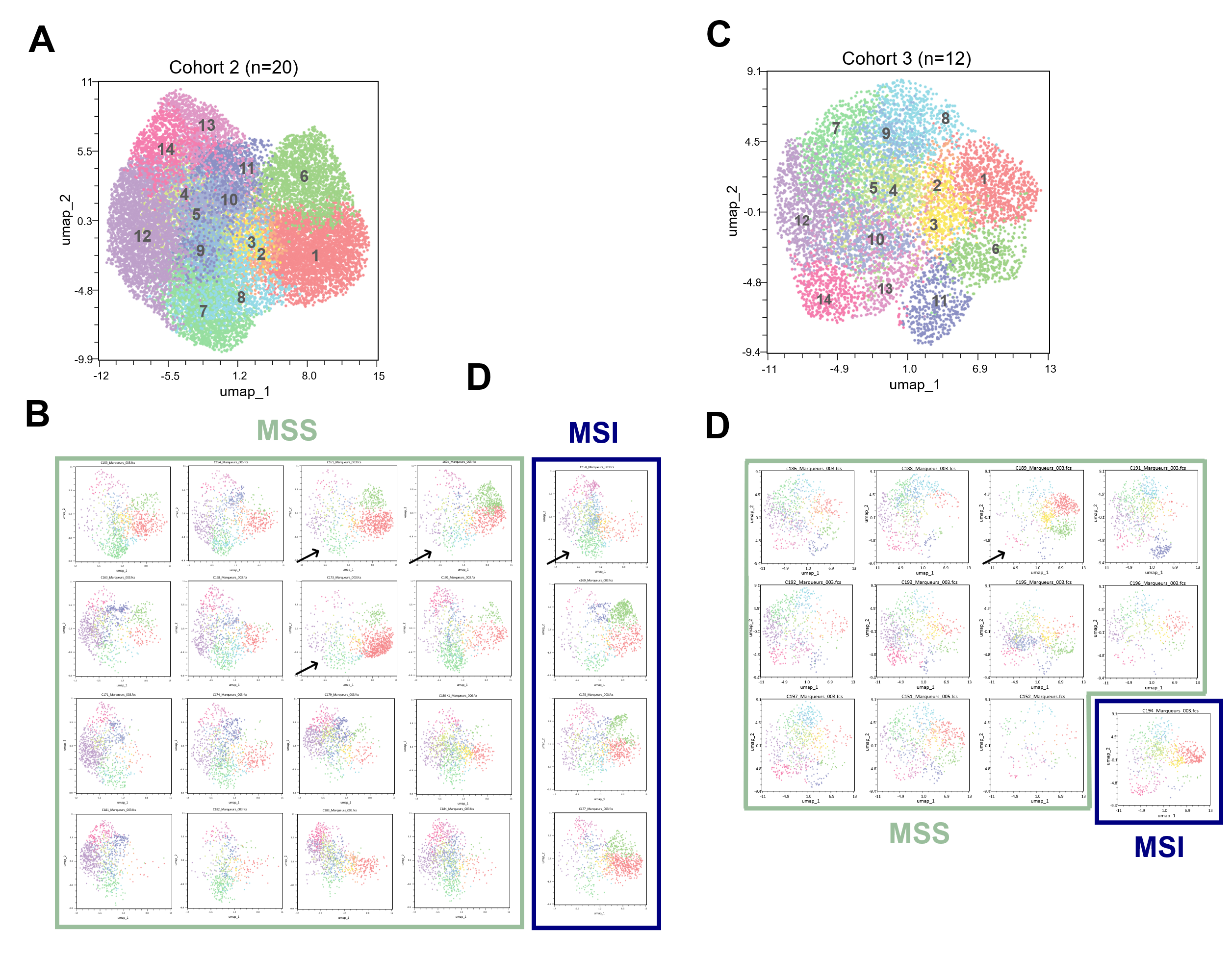

Supplement: Supplementary file 1 [file cancers-14-04261-s001.zip › Figure S6.tif]
